# Fragmentation reduces regional-scale spatial genetic structure in a wind-pollinated tree because genetic barriers are removed

**DOI:** 10.1002/ece3.344

**Published:** 2012-08-05

**Authors:** Rong Wang, Stephen G Compton, Yi-Su Shi, Xiao-Yong Chen

**Affiliations:** 1 Tiantong National Observation Station of Forest Ecosystems, Department of Environmental Sciences, East China Normal UniversityShanghai, 200062, China; 2 School of Biology, Faculty of Biological Sciences, University of LeedsLeeds, LS2 9JT, UK

**Keywords:** *Castanopsis*, dispersal barrier, fragmentation, genetic structure, pollen flow, wind-pollination

## Abstract

Gene flow strongly influences the regional genetic structuring of plant populations. Seed and pollen dispersal patterns can respond differently to the increased isolation resulting from habitat fragmentation, with unpredictable consequences for gene flow and population structuring. In a recently fragmented landscape we compared the pre- and post-fragmentation genetic structure of populations of a tree species where pollen and seed dispersal respond differentially to forest fragmentation generated by flooding. *Castanopsis sclerophylla* is wind-pollinated, with seeds that are dispersed by gravity and rodents. Using microsatellites, we found no significant difference in genetic diversity between pre- and post-fragmentation cohorts. Significant genetic structure was observed in pre-fragmentation cohorts, due to an unknown genetic barrier that had isolated one small population. Among post-fragmentation cohorts this genetic barrier had disappeared and genetic structure was significantly weakened. The strengths of genetic structuring were at a similar level in both cohorts, suggesting that overall gene flow of *C. sclerophylla* has been unchanged by fragmentation at the regional scale. Fragmentation has blocked seed dispersal among habitats, but this appears to have been compensated for by enhanced pollen dispersal, as indicated by the disappearance of a genetic barrier, probably as a result of increased wind speeds and easier pollen movement over water. Extensive pollen flow can counteract some negative effects of fragmentation and assist the long-term persistence of small remnant populations.

## Introduction

The spatial distribution of genetic variation can provide perspectives into current and past population dynamics, and hence is of great significance for biological conservation (Escudero et al. 2003; Lowe et al. 2005; Storfer et al. 2007). The existence of spatial genetic structure (SGS) decreases effective population size, affecting population genetic diversity and even progeny fitness in the long term (Kalisz et al. 2001; Fenster et al. 2003; Wang et al. 2009). Both theoretical and empirical studies have shown that gene flow plays a critical role in determining the extent of relatedness among adjacent individuals and levels of local random genetic drift, and that gene flow is the predominant determinant of SGS in the absence of selection. SGS in turn is influenced by a range of factors such as mating systems, genetic discontinuities arising from historical events and the extent of seed dispersal (Vekemans and Hardy 2004; Hardy et al. 2006; de-Lucas et al. 2009; Gonzales et al. 2010; Segelbacher et al. 2010).

Habitat fragmentation reduces population sizes and often produces biologically depauperate, geographically isolated remnant patches in fragmented landscapes (Young et al. 1996; Terborgh et al. 2001; Lu et al. 2006). The variably hostile environments surrounding remnant populations are expected to generally represent barriers to gene flow and thus lead to enhanced SGS in fragmented habitats (Hamrick 2004; Van Rossum and Triest 2006; Aguilar et al. 2008; Provan et al. 2008; Eckert et al. 2009). However, contrary to this prediction, some results on SGS patterns in forest remnants suggest that gene flow can be unaffected or even enhanced by fragmentation (Dick et al. 2003; Williams et al. 2007; Born et al. 2008; Bizoux et al. 2009). This discrepancy may suggest a continuum of gene flow responses to fragmentation which depends on the mechanisms whereby genes are dispersed and how they respond to the environmental conditions separating remnant patches in fragmented landscapes (de-Lucas et al. 2009; Jaquiéry et al. 2011).

Gene flow in plants is mediated mainly by effective pollen and seed establishment (Chen et al. 2008). Small habitat patches, increased spatial isolation and changes in the environment between patches can impact seed and pollen dispersal between remnant plant populations in different ways. For plants that depend on vertebrates for long-distance seed dispersal, the extinction or reduced abundance of birds, bats, or other suitable seed vectors in remnant patches reduces opportunities for dispersal (Cordeiro and Howe 2003; Uriarte et al. 2011). Increased spatial isolation generates further barriers to dispersal if the patches are surrounded by hostile environments that are unsuitable for both the plants and their remaining dispersal agents (Wunderle 1997; Ghazoul 2005). Seed dispersal therefore tends to become more restricted and concentrated within fragments, an effect that is especially strong in species with large seeds dispersed by terrestrial mammals, because these animals are particularly sensitive to fragmentation (Ghazoul 2005; Bittencourt and Sebbenn 2007; Cramer et al. 2007). Changes in fine-scale SGS can be generated by such alterations in seed dispersal, even if pollen flow is unaffected, in part because each seed disperses two copies of each genes, but pollen grains contain only one (Bittencourt and Sebbenn 2007; Wang et al. 2011).

Pollen dispersal is generally less impacted by fragmentation than seed dispersal (e.g., White et al. 2002; Lowe et al. 2005; Born et al. 2008; Wang et al. 2011). This partly reflects the greater resilience and diversity of insects (the main group of biotic pollen vectors) compared with vertebrates (the major seed dispersers) (Ewers and Didham 2006), and wind-pollinated plant species also appear particularly resistant to fragmentation, with little evidence of it resulting in decreased genetic diversity, increased genetic differentiation, and higher rates of inbreeding (Williams et al. 2007; Kramer et al. 2008; Bizoux et al. 2009). Furthermore, it has been reported that fragmentation can enhance pollen dispersal in some wind-pollinating trees, possibly because of more rapid and unimpeded movements of air current in open areas (Bacles et al. 2005; Sork and Smouse 2006). Reflecting this, relative to seed dispersal, pollen movement plays a more critical role in genetically connecting populations into stepping stone networks in fragmented landscape, thereby potentially influencing SGS at larger spatial scales (Petit et al. 2005; Lander et al. 2010). Consequently, fragmentation can potentially weaken regional-scale SGS if it increases the extent of pollen movements, even if fragmentation has simultaneously reduces seed dispersal distances.

*Castanopsis sclerophylla* (Fagaceae) is a widely distributed monoecious canopy tree of subtropical evergreen forests in southeastern China. It can reach a height of about 25 m, is wind-pollinated, and flowers in April and May. In November, the heavy nuts of *C. sclerophylla* fall to the ground, from where there is some secondary dispersal by rodents. Previous studies of populations of this species in Qiandao Lake, a fragmented landscape of numerous islands formed when a river was dammed about 50 years ago, have shown that seed dispersal is restricted to within remnant habitat islands, with most seeds being dispersed less than 30 m from their maternal parents (R. Wang, unpublished data). Conversely, pollen flow may be more extensive across the water separating the plant populations than it is in the forest patches, because a large pollen-to-seed migration ratio (162) was estimated for plants within an area of 100 km^2^ encompassing the lake (Zhang et al. 2012). Fragmentation has resulted in strengthened SGS among younger post-fragmentation trees at fine spatial scales, due to more localized seed dispersal, but extensive pollen dispersal between fragments appears to have prevented any changes in the extent of genetic differentiation between the pre- and post-fragmentation cohorts (Wang et al. 2011).

The post-fragmentation changes in SGS we detected at small geographical scales may not necessarily be reflected regionally, because of the predominant role of pollen dispersal in shaping SGS at larger spatial scales. Here, we compare SGS among pre- and post-fragmentation cohorts of *C. sclerophylla* to evaluate the impact of habitat fragmentation on pollen dispersal under circumstances where seed dispersal is known to be strongly restricted. Specifically, we address the following questions: (1) Is there a significant difference in genetic structure between pre- and post-fragmentation cohorts at the landscape scale? And (2) based on any changes in landscape genetic patterns, what can be inferred about the effects of fragmentation on patterns of pollen flow in *C. sclerophylla*?

## Material and Methods

### Study area and sampling scheme

**Figure 1:**
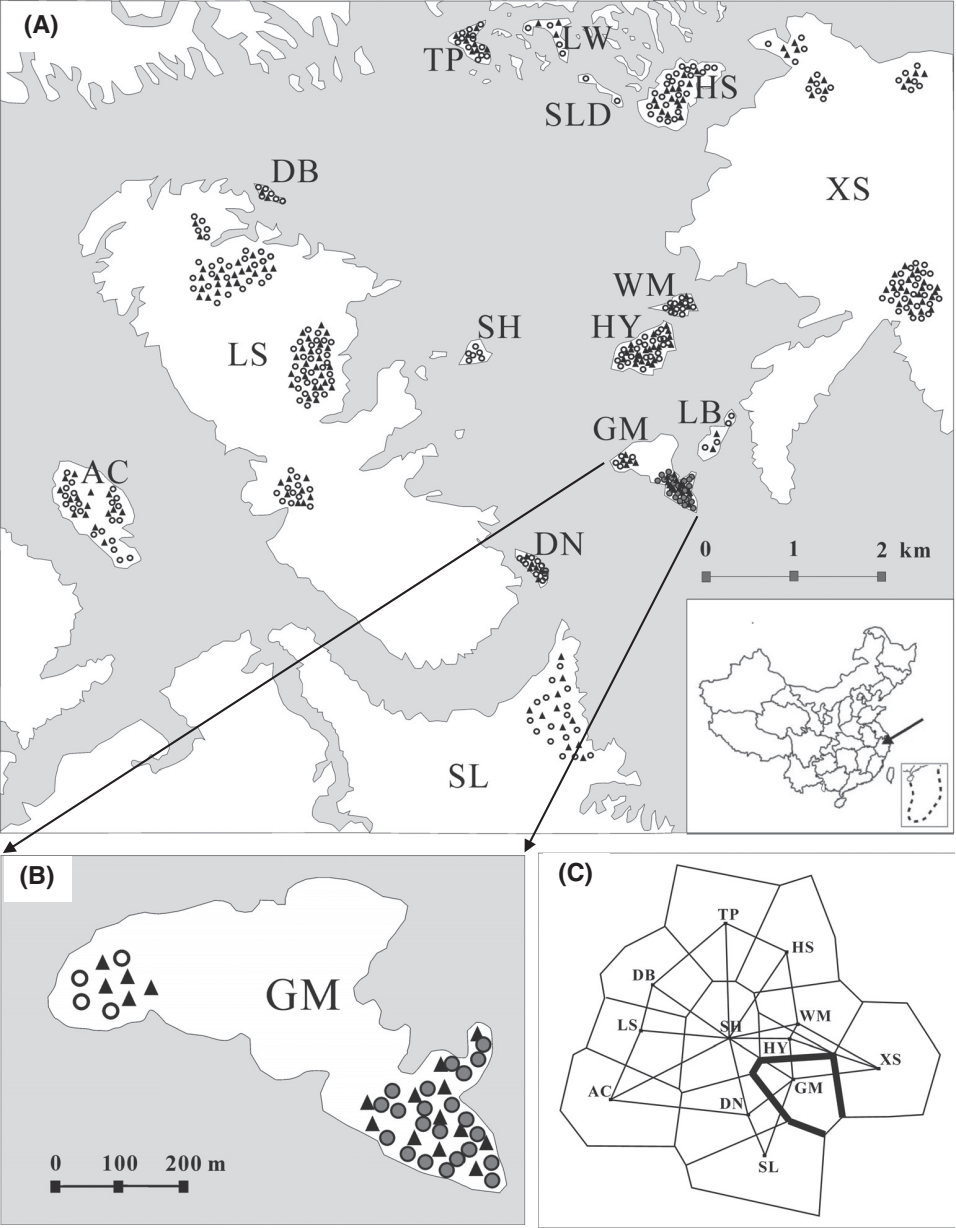
(A) The positions of populations and sampled individuals in the southeast sub-region of Qiandao Lake. Circles and filled triangles represent pre- and post-fragmentation samples, respectively (many points represent multiple individuals). Inset: the location of the study area within China. (B) Two genetic sub-groups were identified among pre-fragmentation individuals using Geneland, one of which was restricted to population GM. The 22 individuals that were recorded as distinct are indicated by gray circles. (C) Bold lines in the attached map on the left side represented the genetic barrier revealed by BARRIER, which also segregated GM from other pre-fragmentation subpopulations.

This study was conducted in and around Qiandao Lake (N29°30.00′–N29°35.00′, E119°00.00′–E119°07.50′), a hydroelectric impoundment formed in 1959 by the establishment of Xin'anjiang Dam. When the dam is full, the lake contains 1078 islands with an area larger than 2500 m^2^. Before the lake was formed, forests were more or less continuous in the area. Currently, *C. sclerophylla* is one of the dominant trees in forests of the southeastern part of the lake, but would have been more widespread previously. According to field observations (Zhang et al. 2007; R. Wang, unpublished data), it is now mainly distributed in forest patches on thirteen islands and two areas of adjacent continuous forests (Fig. 1A, Table 1). Twelve small and three large populations were examined, with *C. sclerophylla* population sizes ranging from three to >35,000 individuals (Table 1). The distances between pairs of populations ranged from 100 m to about 8 km (Fig. 1A). All our study populations, other than population SL, were located to the north of the Xin'anjiang River, which ran through the area prior to impoundment (Fig. 1A). The populations can be categorized into two groups according to their population sizes: small seriously fragmented populations found on islands covering less than 100 ha that contained fewer than 500 individuals, and much larger and less fragmented populations from around the lake and on a large island (LS) with an area of about 875 ha.

**Table 1:** Genetic diversity based on eight microsatellite loci from 15 populations of *Castanopsis scerophylla*

					Pre-fragmentation						Post-fragmentation					
						
Code	Population	Population size	Area (ha)	Location	*N*	*A*	*A*_R_	*H*_O_	*H*_E_	*F*_IS_	*N*	*A*	*A*_R_	*H*_O_	*H*_E_	*F*_IS_
Small populations																
AC	Aci	400	50.6	N 29˚31.87′, E 119˚02.72′	21	5.50	3.82	0.55	0.62	0.126	15	5.50	4.10	0.67	0.67	0.002
DB	Dongbei	14	0.6	N 29˚33.66′, E 119˚03.46′	6	4.00	4.00	0.60	0.65	0.071	2	NA	NA	NA	NA	NA
DN	Dongnan	32	1.1	N 29˚31.63′. E 119˚05.17′	11	5.00	4.16	0.56	0.67	0.180	6	3.75	3.75	0.52	0.67	0.235
GM	Guanmiao	100	15.6	N 29˚32.19′, E 119˚05.97′	26	5.25	3.51	0.44	0.56	0.220*	17	4.88	3.75	0.46	0.64	0.287*
HS	Huangshan	500	39.8	N 29˚34.19′, E 119˚05.86′	23	6.00	4.09	0.59	0.66	0.108	15	5.38	4.32	0.65	0.70	0.080
HY	Heyang	350	13.0	N 29˚32.84′, E 119˚05.91′	22	5.75	3.99	0.59	0.64	0.090	21	5.50	3.75	0.51	0.62	0.179
LB	Longbao	6	6.9	N 29˚32.34′, E 119˚06.34′	4	NA	NA	NA	NA	NA	2	NA	NA	NA	NA	NA
LW	Lianwan	7	3.9	N 29˚34.61′, E 119˚05.23′	4	NA	NA	NA	NA	NA	3	NA	NA	NA	NA	NA
SH	Shihu	6	1.5	N 29˚32.81′, E 119˚04.82′	6	4.25	4.25	0.52	0.66	0.221	–	–	–	–	–	–
SA	Sanlian	3	0.5	N 29˚34.49′, E 119˚05.46′	2	NA	NA	NA	NA	NA	–	–	–	–	–	–
TP	Taiping	22	4.5	N 29˚34.64′, E 119˚04.77′	12	4.88	4.05	0.55	0.64	0.147	8	4.00	3.73	0.50	0.61	0.196
WM	Wuming	21	3.2	N 29˚33.06′, E 119˚06.05′	12	4.00	3.47	0.57	0.61	0.069	4	NA	NA	NA	NA	NA
Large populations																
LS	Laoshan	12000	874.9	N 29˚32.95′, E 119˚03.27′	65	7.88	4.02	0.55	0.63	0.118*	50	7.38	4.06	0.60	0.65	0.074
SL	Shilin	7000	885.5	N 29˚31.00′, E 119˚05.48′	16	5.00	3.78	0.54	0.60	0.103	11	4.63	3.90	0.54	0.62	0.139
XS	Xianshan	35000	>2500	N 29˚32.35′, E 119˚07.51′	42	7.50	4.28	0.55	0.63	0.133*	33	7.25	4.19	0.51	0.64	0.210*

*N*: sample size; *A*: average number of alleles per locus; *A*_R_: average allelic richness per locus in a sample of six individuals; *H*_O_: observed heterozygosity; *H*_E_: expected heterozygosity; *F*_IS_: fixation index; –: no individual; NA: not available because the subpopulation size was less than 5.

* *P*<0.05

Sampling of all 15 populations was carried out in the winter of 2009 and the spring of 2010. Each *C. sclerophylla* individual was allocated into either a post- (age <50 years) or pre-fragmentation (age >50 years) cohort based on a previously established relationship between age and basal trunk diameter (Zhang 2006; Wang et al. 2011). Almost all individuals in small populations whose habitat sizes were smaller than 10 ha were censused (Table 1). Elsewhere, the minimum interval between trees for haphazard sampling in the larger populations was set as at least 20 m, because our previous study at fine-scale revealed a strong genetic autocorrelation among trees within this range (Wang et al. 2011). Sampling took place throughout the area of distribution of each *C. sclerophylla* population, but a minimum distance of 20 m from forest edges was maintained, to avoid possible edge effects. Saplings shorter than 1.5 m and trees infested by termites were also excluded, because they often had few leaves. Each sampled individual was mapped by a portable GPS, and several healthy leaves were collected and dried with silica gel for DNA extraction. The total sample size was 459 trees, with 272 and 187 individuals for pre- and post-fragmentation cohorts, respectively (Table 1).

### DNA extraction and microsatellite genotyping

Genomic DNA extraction used a plant genomic DNA kit (Tiangen, Beijing, China). Eight polymorphic microsatellite loci developed in *Castanopsis cuspidata* var. *sieboldii* were selected (Wang et al. 2011). Among these loci, four (Ccu16H15, Ccu17F15, Ccu28H18, and Ccu33H25) were from Ueno et al. (2000), and the rest (Ccu62F15, Ccu87F23, Ccu93H17, and Ccu102F36) were from Ueno et al. (2003). Polymerase chain reactions (PCRs) were carried out to amplify microsatellites with fluorescently labeled forward primers (FAM, HEX, ROX, and TAMRA) on a PTC-220 DNA Dyad thermal cycler (MJ Research, Waltham, MA, USA). Then, the PCR products of multiple loci with different fluorescent labels or nonoverlapping allele distribution ranges were pooled, and fragment lengths of alleles were distinguished on an ABI 3130 Genetic Analyzer (Applied Biosystem, Foster City, CA, USA) using GeneScan500(-250) Liz standard. Scoring of genotypes was carried out using the software GENEMAPPER 4.0 (Applied Biosystem). Note that 40, 42, and 43 individuals were collected in the sampling plots in XS, LS, and HY, respectively, and therefore these samples shared the genotype data with the previous study (Wang et al. 2011).

### Analyses of genetic variation and spatial genetic structure

Departures from Hardy–Weinberg equilibrium (HWE) were tested, and observed (*H*_O_) and expected (*H*_E_) heterozygosities in each pre- or post-fragmentation subpopulation were estimated using GenAlEx version 6.3 (Peakall and Smouse 2006). Average number of alleles per locus (*A*), allele richness (*A*_R_) (El Mousadik and Petit 1996) and fixation index (*F*_IS_) were quantified with FSTAT version 2.9.3.2 (Goudet 1995). Micro-Checker version 2.2.3 (Van Oosterhout et al. 2004) was used to check for the presence of null alleles, assuming HWE. Within each cohort, genetic differentiations were measured by the statistics *F*_ST_ and *G*_ST_ for global, large, and small populations and tested using FSTAT. Genetic differentiation between both cohorts in small and large populations was also estimated, as was standardized genetic differentiation (*F′*_ST_ and *G′*_ST_) as described by Hedrick (2005). When estimating *F′*_ST_, RecodeData version 0.1 (Meirmans 2006) was implemented to calculate the maximum *F*_ST_. Historical gene flow (*Nm*) among subpopulations within each cohort was evaluated according to the formula proposed by Slatkin and Barton (1989). Subpopulations with sample sizes smaller than five were excluded from these analyses.

We also characterized spatial genetic structures in pre- and post-fragmentation cohorts of large, small, and global populations using SPAGeDi version 1.3 (Hardy and Vekemans 2002). The kinship coefficient *F*_ij_ (Loiselle et al. 1995) was used. We divided pairs of individuals in each cohort from large, small, and the overall populations into 8, 9, and 10 spatial separation distances, respectively (1–900 m, 901–1800 m etc.). Tests of significance of SGS were carried out against 9999 random permutations. Intensity of SGS in each autocorrelation analysis was evaluated by the statistic *Sp* using SPAGeDi (Vekemans and Hardy 2004). Heterogeneity tests of correlograms were performed using GenAlEx version 6.3 (Peakall and Smouse 2006). The nonparametric approach described by Smouse et al. (2008) was applied, in which permutations from a pooled dataset were executed to compare the observed differentiation among different correlograms, with randomized ones using Fisher's combined probabilities as a gauge for statistical comparison. The null hypothesis was that no heterogeneity existed among correlograms at any distance interval, with the criteria *ω* and *t*^2^ used to represent the extent of divergences among whole correlograms and separately for each distance interval. The number of bootstraps was set to 9999.

### Detection of genetic discontinuities

We conducted assignment analyses on the pre- and post-fragmentation cohorts using GENELAND version 3.2.4 in R (Guillot et al. 2005a). GENELAND adopts a Bayesian-based approach that considers the number of clusters as a variable estimated by Markov Chain Monte Carlo (MCMC) procedures (Guillot et al. 2005b; Coulon et al. 2006). For samples from each cohort, five runs were implemented independently in the spatial D-model with 100,000 MCMC iterations. Allele frequencies were assumed to be uncorrelated, as recommended by Guillot et al. (2005b) and the null allele model was chosen because null alleles may exist at some loci (Guillot et al. 2008).

If more than one cluster was detected, the geographic distribution of each cluster was established by running GENELAND with a fixed cluster number. Analysis of molecular variance (AMOVA) using GenAlEx version 6.3 (Peakall and Smouse 2006) was used to calculate the proportion of genetic variance within and among different clusters. The significance of this proportion was tested with 9999 permutations. Monmonier's maximum difference algorithm was implemented to detect genetic barriers among subpopulations in cohorts that contained more than one genetic cluster, using Barrier v2.2 (Manni et al. 2004). The robustness of genetic barriers was estimated by 1000 bootstrapped matrices of D_A_ genetic distances (Nei et al. 1983) using Microsatellite analyzer (Dieringer and Schlötterer 2003).

## Results

### Genetic diversity and spatial genetic structure

All eight loci were independent from each other and revealed a high level of polymorphism. The number of alleles per locus varied from 3 to 19 with a mean of 10.88. Significant departures from HWE were detected in populations GM and XS, based on average fixation index values across the eight loci. Pre-fragmentation subpopulation LS and post-fragmentation subpopulation HY were also not at HWE (Table 1). Significant indications of null alleles were found in *Ccu93H17* and *Ccu102F36*, with frequencies of 17.8% and 22.9%, respectively. Note that all the analyses were also calculated excluding the data from *Ccu93H17* and *Ccu102F36*, and the results of six loci were similar to that of eight loci (See Data S1 for details). Thus, we show the results of eight loci. Genetic variation within both cohorts were similar, with no significant differences in allelic richness (*A*_R_), observed (*H*_O_) and expected (*H*_E_) heterozygosity between cohorts (Table 1, *t*-test for *A*_R_: *P* = 0.988; for *H*_O_: *P* = 0.992; for *H*_E_: *P* = 0.246).

Values of *F′*_ST_ and *G′*_ST_ among post-fragmentation subpopulations (0.054 and 0.095, respectively) were much lower than among pre-fragmentation subpopulations (0.111 and 0.138, respectively), with estimated gene flow among post-fragmentation subpopulations (4.355) twice that of pre-fragmentation ones (2.012). In the pre-fragmentation cohort, genetic differentiations (pairwise *F′*_ST_) between subpopulation GM and the other subpopulations (except for DB) were significantly larger than random (Table 2, upper triangle) and when population GM was excluded there was a major decline in the *F′*_ST_ (0.044) and *G′*_ST_ (0.055) of the pre-fragmentation cohort as a whole (*Nm* = 5.438).

**Table 2:** Genetic divergence (*F′*_ST_) between pairs of pre- (upper triangle) and post- (lower triangle) fragmentation subpopulations of *Castanopsis scerophylla*

	Small populations									Large populations		
		
	AC	DB	DN	GM	HS	HY	SH	TP	WM	LS	SL	XS
AC	–	0.007	0.076	0.359*	0.006	0.052	0.106	−0.037	0.074	0.012	0.042	0.063
DB	NA	–	−0.006	0.271	−0.003	0.042	0.036	−0.048	0.009	0.031	0.019	0.018
DN	0.133	NA	–	0.241*	0.018	0.075	−0.088	−0.033	0.084	0.029	0.039	0.048
GM	0.082	NA	0.043	–	0.339*	0.374*	0.261	0.275*	0.378*	0.346*	0.389*	0.283*
HS	−0.009	NA	0.004	0.073	–	0.053	0.030	−0.021	0.031	0.021	0.002	0.040
HY	0.006	NA	0.044	0.139*	0.004	–	0.048	0.030	0.053	0.046	0.078	0.158*
SH	NA	NA	NA	NA	NA	NA	–	−0.014	0.060	0.066	0.131	0.077
TP	0.036	NA	0.100	0.133	0.055	0.020	NA	–	0.064	−0.010	0.018	0.027
WM	NA	NA	NA	NA	NA	NA	NA	NA	–	0.045	0.089	0.099*
LS	−0.011	NA	0.112	0.131*	0.011	0.039	NA	0.059	NA	–	0.020	0.058*
SL	0.046	NA	0.151	0.137	0.026	0.079	NA	0.133	NA	0.049	–	0.059*
XS	0.037	NA	0.070	0.105*	0.037	0.052	NA	0.048	NA	0.041*	0.025	–

NA, not available because the subpopulation size was less than 5.

* *P*<0.05

There was no clear difference in genetic differentiation between the two cohorts from large populations (where the pre-fragmentation cohort *F′*_ST_ = 0.048 and *G′*_ST_ = 0.051; and the post-fragmentation cohort *F′*_ST_ =0.039 and *G′*_ST_ = 0.041), but fragmentation had weakened genetic differentiation among the small populations (pre-fragmentation cohort: *F′*_ST_ = 0.149 and *G′*_ST_ = 0.145; post-fragmentation cohort: *F′*_ST_ = 0.058 and *G′*_ST_ = 0.082). The exclusion of pre-fragmentation subpopulation GM generated a sharp reduction in *F′*_*ST*_ and *G′*_ST_ values among the small populations (pre-fragmentation cohort: *F′*_ST_ = 0.031 and *G′*_ST_ = 0.038). Homogenous patterns of genetic differentiation were detected between groups of large and small populations in both cohorts (in the pre-fragmentation cohort: *F′*_ST_ = 0.028 and *G′*_ST_ = 0.023; in the post-fragmentation cohort: *F′*_ST_ = 0.023 and *G′*_ST_ = 0.019).

**Figure 2:**
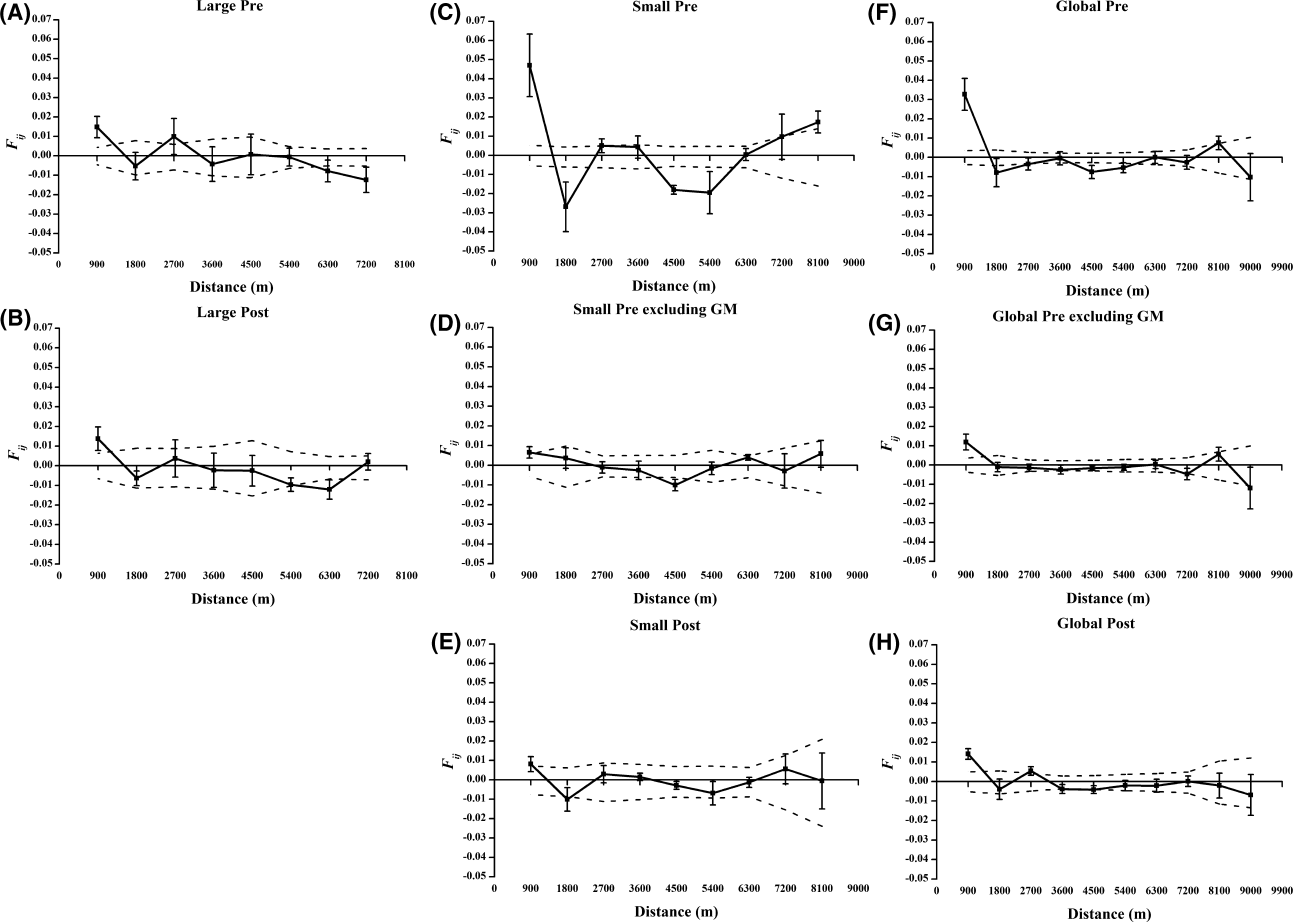
Correlograms of pre- and post-fragmentation cohorts (A–H) from large, small, and global populations from spatial autocorrelation analysis using SPAGeDi. Dashed lines indicate 95% confidence intervals, assuming the null hypothesis that no autocorrelation exists, based on 9999 permutations.

All correlograms showed significantly positive autocorrelations in the first distance interval (≤900 m). Among the large populations, values of the autocorrelation index in the first distance interval were at similar levels in pre- and post-fragmentation cohorts (Fig. 2). In contrast, values of *F*_ij_ in the first distance class for the post-fragmentation cohorts of the small and global populations were just 17.2% and 43.1% of those in their respective pre-fragmentation cohorts (Fig. 2). Emphasizing the distinctive nature of subpopulation GM, its exclusion resulted in sharp decreases in the first distance interval *F*_ij_ values of the pre-fragmentation correlograms (by 86.2% and 63.6%) in the small and global populations, respectively.

Significantly negative *b*_log_ was present in all correlograms (Table 3). Similar values of *Sp* were detected in pre- and post-fragmentation cohorts from large populations (Table 3), but values of *Sp* for pre-fragmentation cohorts were four and two times larger than those of post-fragmentation cohorts from the small and global populations, respectively. When pre-fragmentation subpopulation GM was excluded, these differences in intensity of SGS were absent.

**Table 3:** Values of statistic *Sp* and the slopes of linear regression functions in correlograms analyzed by SPAGeDi

	Large		Small			Global		
			
	Pre	Post	Pre	Pre excluding GM	Post	Pre	Pre excluding GM	Post
*Sp*	0.0060	0.0046	0.0152	0.0029	0.0036	0.0103	0.0043	0.0046
*b*_log_	−0.0059*	−0.0046*	−0.0145*	−0.0029*	−0.0036*	−0.0099*	−0.0043*	−0.0045*

* *P*<0.05.

Homogeneous correlograms (*P* = 0.20) were found between pre and post cohorts from large populations, whereas significant (*P* < 0.05) heterogeneity was present between pre- and post-fragmentation cohorts among small and global populations (both total and several distance intervals of correlograms including the first distance interval; Table 4). Excluding pre-fragmentation subpopulation GM resulted in no significant differentiation in these two pairs of correlograms (*P* = 0.46 and 0.47 in pairwise comparisons for small and global populations, respectively) (Table 3).

**Table 4:** Heterogeneity tests of SGS for pairwise comparisons between pre- and post-fragmentation correlograms in large, small, and global populations using GenAlEx. Statistics *t*^2^ and *ω* represent, respectively, the extent of differentiation of SGS between pairwise correlograms in each distance interval and overall

	Distance class (*t*^2^)										
		
Population groups	1	2	3	4	5	6	7	8	9	10	Total (ω)
Large	0.05	0.01	0.83	0.10	0.27	0.85	0.98	9.30*			20.41
Small	31.83*	7.32*	0.10	0.12	7.05*	4.30*	0.06	0.28	1.38		50.76*
Small, excluding GM Pre	0.09	2.99	0.29	0.53	2.07	0.46	0.81	0.68	0.18		17.87
Global	20.58*	1.04	7.30*	1.44	1.64	1.21	0.40	0.44	1.55	0.24	46.02*
Global, excluding GM Pre	0.33	0.27	4.19*	0.22	0.73	0.02	0.56	1.50	0.95	0.47	19.81

* *P*<0.05.

### Detection of genetic barriers

When the number of clusters was specified as unknown, five independent runs of GENELAND consistently suggested that two genetic clusters were present in the pre-fragmentation cohort. After five further runs with a fixed cluster number, 22 of 26 individuals from subpopulation GM were assigned to a distinct genetic cluster that was significantly different from all remaining individuals of the pre-fragmentation cohort (Fig. 1B). Variance between the two clusters explained a significantly high proportion of the total variance (22.31%, *P* < 0.001), indicating the presence of a genetic discontinuity and two recognizably distinct groups of plants within the overall population. Using the software BARRIER, we confirmed a significant genetic barrier present around pre-fragmentation subpopulation GM (Fig. 1C), with average support of 75.1% in the bootstrapped matrices (varying from 53.7% to 95.4% with different boundaries).

No such barrier was observed among post-fragmentation cohorts. Results from five independent runs of GENELAND converged and inferred that only one genetic cluster was present.

## Discussion

### Effects of habitat fragmentation on regional-scale SGS

Changes in spatial genetic structure of pre- and post-fragmentation cohorts of *C. sclerophylla* were detected among small (and global) populations, with reduced SGS in the post-fragmentation cohort, although no difference was detected in large populations. Values of *F′*_ST_ and *G′*_ST_ among pre-fragmentation subpopulations were much larger than those among post-fragmentation subpopulations in the former two population groups, and spatial autocorrelation analyses also showed significant differences between pre- and post-fragmentation subpopulations at distances ≤900 m and at the global population level. Further analyses indicated that the post-fragmentation reduction in SGS resulted from the removal of a pre-fragmentation genetic barrier that existed around one small island (GM). The overall SGS was therefore not significantly changed by fragmentation in the absence of population GM, indicating that extensive gene flow persisted among remnant populations. This was consistent with some previous studies (e.g., Williams et al. 2007; Kramer et al. 2008; Bizoux et al. 2009). The post-fragmentation disappearance of the genetic barrier can be attributable to the tree's pollen dispersing for longer distances over water than across what previously would have been continuous forest (White et al. 2002; Dick et al. 2003; Sork and Smouse 2006; Llorens et al. 2012). The extent of seed dispersal, in contrast, has been strongly limited by rise in water levels (Wang et al. 2011). The significant alteration in the genetic composition of population GM suggested that pollen flow had been enhanced by fragmentation.

Genetic barriers are often observed within the ranges of plant species, even when they occupy a continuous habitat, as a result of historical events such as previous fragmentation and recolonization (Born et al. 2008; Debout et al. 2011), but lag times after genetic barriers are removed are highly variable in duration (Landguth et al. 2010). In *C. sclerophylla*, we found that a previous genetic dispersal barrier has disappeared within one to two generations of its removal and is now only evident among the oldest individuals. This loss of a genetic barrier has occurred at the same time as genetic diversity has been maintained, and is consistent with the conclusion that gene flow among wind-pollinated trees can be stimulated by habitat fragmentation (Bacles et al. 2005; Williams et al. 2007; Bizoux et al. 2009; but see Jump and Peñuelas 2006). We cannot, however, confirm the precise mechanism leading to the establishment of the genetic barrier, due to lack of historical information of local populations. One possible scenario could be that population GM was located on the northwest boundary of a particular genetic cluster which is distinguished from the unit containing all other populations in the present study. After fragmentation, all individuals of this genetic cluster could have been destroyed by the flood except for GM (no *C. sclerophylla* individual was found on islands in the southeast area of our study range), and then increased pollen flow carrying pollen from the other cluster to population GM rapidly changed its genetic composition, leading to the removal of the genetic barrier.

As in a previous study (Wang et al. 2011), we found similar levels of genetic diversity in pre- and post-fragmentation subpopulations, a result that is consistent with other studies suggesting that post-fragmentation diversity loss is chronic rather than rapid in tree populations (Young et al. 1996; Lowe et al. 2005; Aguilar et al. 2008). Given that Qiandao Lake has been formed for only 53 years, equivalent to one or two generations for *C. sclerophylla*, genetic drift has not had sufficient time to significantly affect genetic variation.

### Different effects of fragmentation on pollen and seed dispersal

Dispersal processes in fragmented landscapes, and consequently the extent of connectivity between remnant patches, are strongly influenced by the nature of the matrix that separates them (Ricketts 2001; Vignieri 2005), to the extent that matrix characteristics can more strongly dictate ecological responses to fragmentation than patch configuration and separation (Swift and Hannon 2010). In general, the more similar the matrix to the habitat fragments, the lower the impact of fragmentation, because migration between patches is less likely to be disrupted (Pulliam 1988). Open-water areas such as those of Qiandao Lake are totally different in character to the original forest that is often thought to greatly reduce inter-fragment movements by terrestrial organisms (e.g., Terborgh et al. 2001). Reflecting this, our previous study detected increased post-fragmentation SGS in remnant populations of *C. sclerophylla* because of severely restricted seed dispersal by rodents (Wang et al. 2011).

The reduction in gene flow via seed dispersal in response to fragmentation has not been reflected in similar reductions in pollen dispersal. To the contrary, we have now detected more extensive pollen dispersal in the post-fragmentation cohort. These contrasting genetic responses to the water matrix surrounding the remnant populations reflect the different interactions between the specific pollen and seed dispersal methods of *C. sclerophylla* (wind-pollination and secondary seed dispersal by small mammals) and the characteristics of this particular anthropogenic disturbance. The open-water area has largely or entirely prevented cross-island migration by the rodents that act as secondary seed dispersers, but has also extended the distances that pollen grains are carried. This can be contrasted with species such as *Fraxinus excelsior* that rely on wind for both seed and pollen dispersal, which is not negatively affected by fragmentation (Bacles et al. 2006). Similarly, plants with seeds that are dispersed by birds and fruit bats will be less affected by fragmentation than species like *C. sclerophylla* that depend on nonvolant animals (e.g., Bacles et al. 2004; Mathiasen et al. 2007).

Landscape genetics methods, such as spatial autocorrelation analysis, are flexible enough to unravel SGS and underlying gene-flow patterns at multiple scales, and so provide insights into genetic responses to changes in landscape configuration (Manel et al. 2003; Escudero et al. 2003; Storfer et al. 2007; Segelbacher et al. 2010). Our empirical studies of *C. sclerophylla* landscape-scale genetics have revealed contrasting patterns of seed and pollen dispersal at different scales, showing that this is a useful tool for measuring genetic consequences at an early stage of habitat fragmentation (Segelbacher et al. 2010).

Extensive gene flow such as we detected in *C. sclerophylla* can reduce or prevent genetic divergence among fragmented populations at regional scales, help maintain overall genetic variation and thereby increase the viability of small remnant populations by reducing the detrimental influences of inbreeding depression and genetic drift (Ellstrand and Elam 1993; Hamrick 2004; Kramer et al. 2008). The increased pollen movement should therefore be beneficial to the small remaining post-fragmentation populations of *C. sclerophylla*. The stabilized SGS at both local and regional scales confirms that genetic variation can be sustained in this kind of remnant population because large effective population sizes are maintained, irrespective of fragmentation (Young et al. 1996; Hamrick 2004).

## Conclusions

Our results suggest that multi-scale landscape genetic studies are necessary to comprehensively understand the genetic consequences of fragmentation, given scale-dependent seed and pollen dispersal, and their differential responses to fragmentation. Fragmentation has different impacts on pollen and seed dispersal in wind-pollinated and gravity- plus-rodent-dispersed trees such as *C. sclerophylla*. No significant difference in genetic diversity was found between pre- and post-fragmentation cohorts. Although restricted seed dispersal strengthened fine-scale spatial genetic structure at local scales (Wang et al. 2011), regional-scale SGS of *C. sclerophylla* was not significantly changed after fragmentation, suggesting consistent gene flow. Furthermore, the disappearance of a pre-fragmentation genetic barrier provides evidence of enhanced pollen dispersal. Even without seed dispersal between fragments, pollen dispersal can link patchy populations and have a positive effect on genetic diversity. This should favor the persistence of remnant populations.

## Supporting Information

**Data S1.** Results excluding loci with null alleles.
