# Supplementary material for: Fragmentation reduces regional-scale spatial genetic structure in a wind-pollinated tree because genetic barriers are removed
Source: Ecol Evol. 2012 Aug 5;2(9):2250–61. doi: 10.1002/ece3.344 (PMC3488675; doi:10.1002/ece3.344)
Supplement: Supplementary file 1 [file ece30002-2250-SD1.docx]

**Results excluding loci with null alleles**

Genetic diversity, differentiation and historical gene flow

All the six loci were proved to be independent from each other and revealed a high level of polymorphism. Number of alleles per locus varied from 3 to 17 with a mean of 8.88. According to values of average fixation index over six loci, significant departure from Hardy-Weinberg equilibrium was not detected in any population (Table 1). Genetic variation within both cohorts were at the same level, and there was no significant difference in allelic richness (*A_R_*), observed (*H_o_*) and expected (*H_e_*) heterozigosity between cohorts (Table 1, *t*-test for *A_R_*: *p*=0.367; for *H_o_*: *p*=0.639; for *H_e_*: *p*=0.525).

Values of *F′_ST_* and *G′_ST_* among post-fragmentation subpopulations (0.0438 and 0.0549 respectively) were much lower than those among pre-fragmentation ones (0.0923 and 0.1186 respectively), and thus, the estimated gene flow among post-fragmentation subpopulations (5.456) was twice more than that among pre-fragmentation ones (2.459). In pre-fragmentation cohort, genetic differentiations (pair-wise *F′_ST_*) between subpopulation GM and most other subpopulations were significantly larger than random cases except for DB, DN, SH and TP(Table 2, upper triangle). When population GM was excluded, dramatic decline of *F′_ST_* (0.0481) and *G′_ST_* (0.0709) were found in pre-fragmentation cohort.

There was no obvious difference in genetic differentiation between two cohorts among large populations (in pre-fragmentation cohort: *F′_ST_*= 0.0557 and *G′_ST_* =0.0536; and in post-fragmentation cohort: *F′_ST_*= 0.0433 and *G′_ST_* =0.0373), whereas fragmentation apparently weakened genetic differentiation among small populations (pre-fragmentation cohort: *F′_ST_*= 0.1100 and *G′_ST_* =0.1133; post-fragmentation cohort: *F′_ST_*= 0.0289 and *G′_ST_* =0.0341). Similarly, with exclusion of pre-fragmentation subpopulation GM, a sharp reduction in values of *F′_ST_* and *G′_ST_* was observed among small populations (pre-fragmentation cohort: *F′_ST_*= 0.0370 and *G′_ST_* =0.0517). Furthermore, homogenous patterns of genetic differentiation were detected between groups of large and small populations in both cohorts (in pre-fragmentation cohort: *F′_ST_*= 0.0337 and *G′_ST_* =0.0293; and in post-fragmentation cohort: *F′_ST_*= 0.0236 and *G′_ST_* =0.0169).

Genetic barrier

When the number of clusters was specified as unknown, five independent runs of GENELAND suggested two genetic clusters in individuals of pre-fragmentation cohort. Furthermore, after five more runs with fixed cluster number, twenty-two out of twenty six individuals in subpopulation GM were assigned to a particular genetic cluster significantly different from the other individuals of pre-fragmentation cohort (completely identical to the result from eight loci). Variance between the two clusters explained significantly high proportion of total variance (14.99%, *p*<0.001), verifying that this barrier truly existed in the study area. Using the software BARRIER, we found a significant genetic barrier around pre-fragmentation subpopulation GM with an average support of 68.6% bootstrapped matrices (varying from 62.0% to 90.7% in different boundaries), confirming the conclusion by GENELAND (identical to the result from eight loci). However, results from five independent runs of GENELAND converged and inferred only one genetic cluster existing in post-fragmentation cohort, indicating that the genetic barrier around GM disappeared after fragmentation.

Difference between correlograms

All correlograms except that of small populations in post-fragmentation cohort showed significantly positive autocorrelation in the first distance interval (≤ 900 m), values of *F_ij_* in small and global populations in post-fragmentation cohort were just 13.5% and 49.2% of those in pre-fragmentation cohort respectively (Fig. 1). Excluding subpopulation GM resulted in sharp decreases of 73.2% and 46.9% in the first distance interval *F_ij_* values of pre-fragmentation correlograms in small and global populations respectively. For large populations, values of autocorrelation index in the first distance interval were at the similar level in pre- and post-fragmentation cohorts (Fig. 1).

Significantly negative *b-log* existed in all correlograms except in that of small populations in post-fragmentation cohort (Table 3). Values of statistic *Sp* of pre-fragmentation cohorts were six and two times larger than those in post-fragmentation ones in small and global populations, respectively. If pre-fragmentation subpopulation GM was excluded, similar intensity of SGS was observed in those two pair-wise comparisons. In large populations, similar values of *Sp* were detected in pre- and post-fragmentation cohorts (Table 3).

Both in small and global populations, significant heterogeneity was found between total correlograms of pre- and post-fragmentation cohorts (*p*<0.05) and at the first distance intervals (Table 4). Significant heterogeneity was also found in two and one more distance intervals in small and global populations respectively. Excluding pre-fragmentation subpopulation GM resulted in no significant differentiation in these two pairs of correlograms (*p*=0.573 and 0.370 in pair-wise comparisons in small and global populations respectively) (Table 4). Homogeneous correlograms (*p*=0.512) were found between pre- and post cohorts in large populations.

Table 1. Genetic diversity over 8 microsatellite loci in each population of *Castanopsis scerophylla*. N: sample size; *A*: average number of alleles per locus; *A_R_*: average allelic richness per locus; *H_O_*: observed heterozygosity; *H_E_*: expected heterozygosity; *F_IS_*: fixation index.

| Code | Population | Pre-fragmentation | | |  |  |  | Post-fragmentation | | |  |  |  |
| --- | --- | --- | --- | --- | --- | --- | --- | --- | --- | --- | --- | --- | --- |
|  |  | N | *A* | *A_R_* | *H_O_* | *H_E_* | *F_IS_* | N | *A* | *A_R_* | *H_O_* | *H_E_* | *F_IS_* |
|  |  |  |  |  |  |  |  |  |  |  |  |  |  |
| AC | Aci | 21 | 4.83 | 3.46 | 0.63 | 0.60 | -0.059 | 15 | 4.50 | 3.62 | 0.73 | 0.64 | -0.148 |
| DB | Dongbei | 6 | 3.83 | 3.83 | 0.69 | 0.65 | -0.068 | 2 | NA | NA | NA | NA | NA |
| DN | Dongnan | 11 | 4.67 | 3.98 | 0.62 | 0.64 | 0.036 | 6 | 3.50 | 3.50 | 0.58 | 0.62 | 0.071 |
| GM | Guanmiao | 26 | 5.00 | 3.45 | 0.51 | 0.55 | 0.078 | 17 | 4.67 | 3.50 | 0.51 | 0.61 | 0.168 |
| HS | Huangshan | 23 | 5.83 | 3.97 | 0.67 | 0.66 | -0.027 | 15 | 4.83 | 3.96 | 0.70 | 0.66 | -0.059 |
| HY | Heyang | 22 | 5.00 | 3.63 | 0.66 | 0.62 | -0.068 | 21 | 4.50 | 3.34 | 0.55 | 0.58 | 0.063 |
| LB | Longbao | 4 | NA | NA | NA | NA | NA | 2 | NA | NA | NA | NA | NA |
| LW | Lianwan | 4 | NA | NA | NA | NA | NA | 3 | NA | NA | NA | NA | NA |
| SH | Shihu | 6 | 4.17 | 4.17 | 0.61 | 0.62 | 0.018 | - | - | - | - | - | - |
| SA | Sanlian | 2 | NA | NA | NA | NA | NA | - | - | - | - | - | - |
| TP | Taiping | 12 | 4.50 | 3.78 | 0.64 | 0.62 | -0.036 | 8 | 3.83 | 3.55 | 0.54 | 0.60 | 0.099 |
| WM | Wuming | 12 | 3.83 | 3.31 | 0.63 | 0.58 | -0.076 | 4 | NA | NA | NA | NA | NA |
|  |  |  |  |  |  |  |  |  |  |  |  |  |  |
| LS | Laoshan | 65 | 7.00 | 3.67 | 0.60 | 0.59 | -0.018 | 50 | 6.00 | 3.63 | 0.66 | 0.61 | -0.084 |
| SL | Shilin | 16 | 4.67 | 3.51 | 0.60 | 0.58 | -0.044 | 11 | 4.33 | 3.74 | 0.65 | 0.63 | -0.041 |
| XS | Xianshan | 42 | 6.67 | 3.97 | 0.57 | 0.59 | 0.039 | 33 | 6.50 | 3.84 | 0.52 | 0.60 | 0.132 |

Table 2. Genetic divergence (*F′_ST_*) in pairs of pre- (upper triangle) and post- (lower triangle) fragmentation subpopulations of *Castanopsis scerophylla*.

|  | Small populations | | | | | | | | | Large populations | | |
| --- | --- | --- | --- | --- | --- | --- | --- | --- | --- | --- | --- | --- |
|  | AC | DB | DN | GM | HS | HY | SH | TP | WM | LS | SL | XS |
| AC | - | 0.063 | 0.056 | 0.277* | 0.020 | 0.069 | 0.130 | -0.029 | 0.080 | 0.012 | 0.074 | 0.075* |
| DB | NA | - | -0.038 | 0.097 | 0.014 | 0.077 | 0.075 | -0.028 | 0.027 | 0.050 | 0.074 | 0.017 |
| DN | 0.058 | NA | - | 0.156 | -0.011 | 0.048 | -0.026 | -0.055 | 0.104 | 0.039 | 0.004 | 0.048 |
| GM | 0.065 | NA | -0.009 | - | 0.241* | 0.241* | 0.149 | 0.173 | 0.285* | 0.273* | 0.294* | 0.236* |
| HS | 0.003 | NA | 0.005 | 0.061 | - | 0.047 | 0.048 | -0.017 | 0.022 | 0.018 | -0.005 | 0.041 |
| HY | -0.008 | NA | -0.032 | 0.057 | -0.006 | - | 0.077 | 0.028 | 0.046 | 0.050 | 0.099 | 0.173* |
| SH | NA | NA | NA | NA | NA | NA | - | -0.003 | 0.131 | 0.109 | 0.151 | 0.099 |
| TP | 0.0546 | NA | -0.018 | 0.063 | 0.074 | 0.012 | NA | - | 0.062 | -0.018 | 0.035 | 0.031 |
| WM | NA | NA | NA | NA | NA | NA | NA | NA | - | 0.050 | 0.099 | 0.099 |
| LS | -0.010 | NA | 0.065 | 0.128* | 0.028 | 0.009 | NA | 0.051 | NA | - | 0.024 | 0.068* |
| SL | 0.044 | NA | 0.017 | 0.089 | -0.007 | 0.039 | NA | 0.099 | NA | 0.047* | - | 0.063 |
| XS | 0.059 | NA | -0.001 | 0.090 | 0.055 | 0.046 | NA | 0.053 | NA | 0.053* | 0.008 | - |

NA: not available because the subpopulation size is less than 5; *: *p*<0.05

Table 3. Values of statistic *Sp* and the slopes of linear regression function in each correlograms analyzed by SPAGeDi.

|  | Large |  | Small |  |  | Global |  |  |
| --- | --- | --- | --- | --- | --- | --- | --- | --- |
|  | Pre | Post | Pre | Pre excluding GM | Post | Pre | Pre excluding GM | Post |
| *Sp* | 0.0073 | 0.0060 | 0.0118 | 0.0040 | 0.0019 | 0.0099 | 0.0055 | 0.0049 |
| *blog* | -0.0071* | -0.0059* | -0.0114* | -0.0040* | -0.0019 | -0.0096* | -0.0054* | -0.0049* |

*: *p*<0.05.

Table 4. Heterogeneity tests of SGS for two pair-wise comparisons between pre- and post-fragmentation correlograms (pre- vs. post) in large, small and global populations using GenAlEx. Statistics *t*^2^ and *ω* represented the extent of differentiation of SGS between pair-wise correlograms in each distance interval and total respectively.

| Population | Distance class (*t^2^*) | | | | | | | | | | Total (ω) |
| --- | --- | --- | --- | --- | --- | --- | --- | --- | --- | --- | --- |
|  | 1 | 2 | 3 | 4 | 5 | 6 | 7 | 8 | 9 | 10 |  |
| Large | <0.01 | <0.01 | 0.13 | 0.06 | 0.01 | 2.72 | 0.15 | 5.90* |  |  | 15.18 |
| Small | 21.85* | 4.59* | 0.22 | 0.01 | 4.64* | 2.22 | 0.13 | 0.05 | 2.12 |  | 42.29* |
| Small, excluding GM Pre | 0.65 | 1.51 | 0.28 | 0.31 | 1.20 | 0.01 | 0.94 | 1.03 | 0.86 |  | 16.29 |
| Global | 13.55* | 0.95 | 4.04* | 0.31 | 1.86 | 0.04 | 0.29 | 0.37 | 2.94 | 0.38 | 38.00* |
| Global, excluding GM Pre | 0.11 | 0.39 | 4.17* | 0.25 | 0.39 | 0.40 | 0.29 | 1.32 | 2.21 | 0.91 | 21.51 |

*: *p*<0.05.


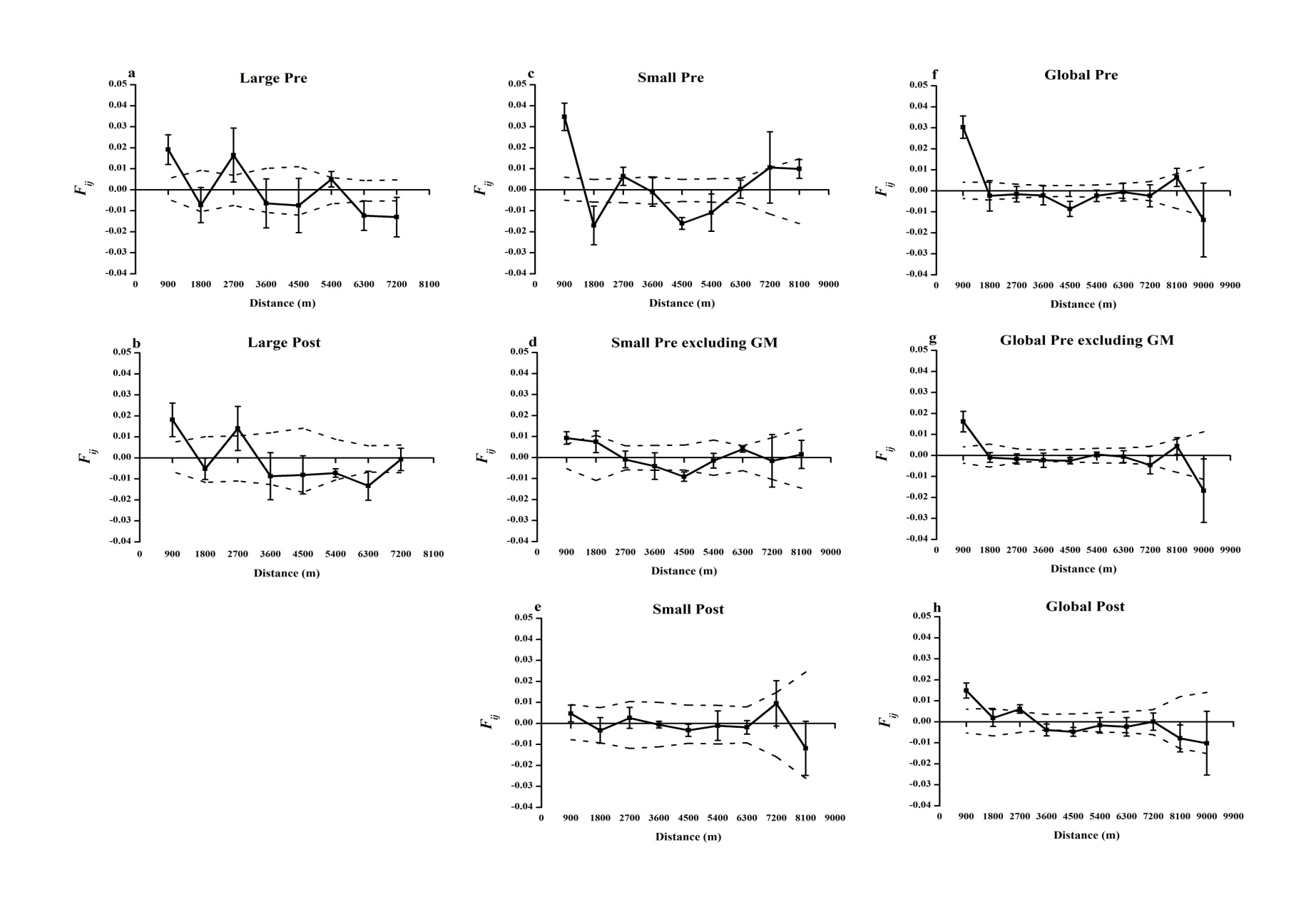


Fig.1. Correlograms from spatial autocorrelation analysis by SPAGeDi in large, small and global populations within pre- and post-fragmentation cohorts (a-h) using GenAlEx.
